# Effects of Meditative Movements on Major Depressive Disorder: A Systematic Review and Meta-Analysis of Randomized Controlled Trials

**DOI:** 10.3390/jcm7080195

**Published:** 2018-08-01

**Authors:** Liye Zou, Albert Yeung, Chunxiao Li, Gao-Xia Wei, Kevin W. Chen, Patricia Anne Kinser, Jessie S. M. Chan, Zhanbing Ren

**Affiliations:** 1 Department of Sports Science and Physical Education, The Chinese University of Hong Kong, Shatin, Hong Kong, China; 2 Depression Clinical and Research Program, Harvard Medical School, Boston, MA 02114, USA; ayeung@mgh.harvard.edu; 3 Department of Health and Physical Education, The Education University of Hong Kong, Tai Po, NT, Hong Kong, China; cxli@eduhk.hk; 4 Key Laboratory of Behavioral Science, Institute of Psychology, Chinese Academy of Sciences, Beijing 100080, China; weigx@psych.ac.cn; 5 Center for Integrative Medicine, School of Medicine, University of Maryland, Baltimore, MD 21201, USA; kchen@umaryland.edu; 6 Department of Family and Community Health Nursing, School of Nursing, Virginia Commonwealth University, Richmond, VA 23298, USA; kinserpa@vcu.edu; 7 Department of Psychology, The University of Hong Kong, Pokfulam, Hong Kong, China; chansm5@hku.hk; 8 Department of Physical Education, Shenzhen University, Shenzhen 518060, China

**Keywords:** mindfulness/meditation, depression, exercise, mood disorders, rehabilitation

## Abstract

Background: Tai Chi, Qigong, and Yoga are recognized as the most popular complementary approaches for alleviating musculoskeletal pain, improving sleep quality, and reducing blood pressure. The therapeutic effects of these meditative movements for treating major depressive disorder (MDD) is yet to be determined. Therefore, we examined whether meditative movements (Tai Chi, Qigong, and Yoga) are effective for treating MDD. Seven electronic databases (SPORTDiscus, PubMed, PsycINFO, Cochrane Library, Web of Science, CNKI, and Wanfang) were used to search relevant articles. Randomized controlled trials (RCT) using Tai Chi, Qigong or Yoga as intervention for MDD were considered for the meta-analysis (standardized mean difference: *SMD*). Results: Meta-analysis on 15 fair-to-high quality RCTs showed a significant benefit in favor of meditative movement on depression severity (*SMD* = −0.56, 95% CI −0.76 to −0.37, *p* < 0.001, *I*^2^ = 35.76%) and on anxiety severity (*SMD* = −0.46, 95% CI −0.71 to −0.21, *p* < 0.001, *I*^2^ = 1.17%). Meditative movement interventions showed significantly improved treatment remission rate (OR = 6.7, 95% CI 2.38 to 18.86, *p* < 0.001) and response rate (OR = 5.2, 95% CI 1.73 to 15.59, *p* < 0.001) over passive controls. Conclusions: Emphasizing the therapeutic effects of meditative movements for treating MDD is critical because it may provide a useful alternative to existing mainstream treatments (drug therapy and psychotherapy) for MDD. Given the fact that meditative movements are safe and easily accessible, clinicians may consider recommending meditative movements for symptomatic management in this population.

## 1. Background

Major depressive disorder (MDD), also known as clinical depression, is one of the most common and debilitating mental illnesses in America [1]. It is estimated that 16.2 million US citizens aged 18 or above had a minimum of one major depressive episode in 2016, representing 6.7% of all American adults [1]. Some common complaints (feeling of worthlessness, low self-esteem, tiredness, impaired cognition, sleep disturbance, recurring thoughts of suicide, and unexplained musculoskeletal pain) persistently present in patients with MDD, leading to reduced quality of life or even mortality [2]. In America, the economic burden of MDD increased by 21.5% between 2005 to 2010, from $173.2 billion to $210.5 billion [3]. The incremental economic burden was attributed to direct costs (e.g., inpatient stay, outpatients visits, rehabilitation, and medications) (45%), loss of productivity (50%), and suicide-related costs (5%) [3]. Such substantial costs not only challenge the national healthcare system, but also place a burden on families of patients with MDD.

The most widely recognized treatments for MDD are pharmacological (antidepressant) therapy and psychotherapy [4]. Apart from the direct and indirect costs of MDD, however, the clinical benefits of these two first-line treatments have been criticized [5,6,7]. When psychotherapy requires time-consuming inputs from health professionals and patients with MDD, use of pharmacological therapy could produce side effects (e.g., sleep disturbance, sexual dysfunction, digestive problems, headache, dizziness, and increased blood pressure) [8]. In addition, previous studies indicated that patients with MDD who took antidepressant medications had poor compliance, high dropout rates, and low remission rates [9,10,11,12].

Given the disadvantages of the first-line treatments with more or less side effects, some researchers recently have shifted their attention to explore the effectiveness of using exercise therapies for treating MDD [13]. As the number of studies grows in this research area, Krogh et al. [14] recently conducted a systematic review and meta-analysis to evaluate the antidepressant effects of non-meditative exercises (cycling, strength training, swimming, jogging, resistance training, and stretching exercise) in patients with MDD, but the aggregated results from the included randomized controlled trials (RCTs) with high-quality traits showed no significant benefit. This finding may give a chance to validate meditative exercises as alternative therapies in the symptomatic management of MDD. Meditative (mind-body) movements, characterized by musculoskeletal stretching and relaxation, breath control, and a meditative state of mind [15,16,17], have been shown to be effective for treating depression, anxiety, and sleep problems in people with mental illness [18,19,20,21]. The National Health Interview Survey reported that meditative movements including Tai Chi, Yoga, and Qigong are ranked as the top three complementary therapies among American adults in workplace [22]. Cramera et al. [23] qualitatively synthesized the effects of Yoga interventions on treating MDD, and no definition conclusion was made. Indeed, there has been an increasing number of well-designed trials showing the positive effects of Tai Chi and Qigong for treating MDD [24,25,26,27,28]. Therefore, a systematic review that critically evaluates the efficacy of the three most popular meditative movements on treating MDD is needed. Findings of this review would provide updated recommendations for researchers and clinicians to design and develop effective meditative movement programs for treating MDD.

## 2. Methods

This review was performed in accordance with the Preferred Reporting Items for Systematic Review and Meta-Analysis (PRISMA) guidelines [29].

### 2.1. Search Strategy

A literature search was conducted by the leading author (L.Z.) of this research, and both Chinese (Wanfang and Chinese National Knowledge Infrastructure; CNKI) and English electronic databases (SPORTDiscus, PsycINFO, PubMed, Cochrane Library, and Web of Science) were searched from their inception to March 2018. Relevant terms were integrated with Boolean conjunction (OR/AND) for search based on three search levels: (i) Tai Chi/Taiji, Yoga, Qigong, mind-body exercise, meditative exercise, meditative movement OR mindful exercise; AND (ii) major depressive disorder, major depression, unipolar depression, OR clinical depression; AND (iii) randomized or randomized controlled trial. Furthermore, reference lists of other reviews and relevant studies were manually searched. 

### 2.2. Eligibility Criteria and Study Selection

The leading review author (L.Z.) initially screened titles and abstracts to remove obviously irrelevant documents and duplicates. This was followed by an examination of abstract and full-text articles, administered by two independent reviewers (L.Z. and C.L.), to determine the eligible studies according to the following inclusion and exclusion criteria. An article was included if it: (i) used a randomized controlled design; (ii) was published in a peer-reviewed journal, (iii) included adult participants diagnosed with MDD based on any valid and clinical diagnostic criteria, (iv) used an intervention that was solely or mainly based on Tai Chi, Yoga, Qigong, or combined mode, (v) had the same co-intervention between meditative movement and control groups, and (vi) reported at least one of primary outcomes (remission rate, response rate, and depressive symptom severity) and secondary outcomes (anxiety severity and sleep quality). Studies investigating two different dosages of a meditative movement for MDD were excluded. Observational studies, case reports/series, controlled trials with no randomization, and review studies were excluded. A third party (A.Y.) was used to resolve disagreements between the two reviewers regarding study selection.

### 2.3. Data Items and Collection Process

Two review authors (L.Z. and C.L.) independently extracted data using a pre-created table. Discrepancies in the data extraction were discussed or resolved by a third reviewer (A.Y.). Data items in the extracted table included references (the first author, year of publication, and country), participant characteristics (initial sample and attrition rate, the number of male/female, mean age/age range, diagnostic criteria, percentage of female, and predominant ethnicity), intervention protocol (weekly training dosage, type of meditative movement, qualification of instructors, total training time, training mode, intervention duration follow-up assessment, and co-intervention), outcome measured, and safety. To calculate the pooled effect size, we also extracted the mean and standard deviation of the outcomes, along with the number of participants in each group.

In order to accurately outline the strengths of each selected study and to correctly interpret the data, we contacted the corresponding author for extra information regarding the details of the study, which may not have been clear in the published paper. We requested the details of 11 selected studies, including the percentage of patients with MDD, eligibility criteria (DSM-IV) administered by psychiatrist(s), percentage of ethnicity, qualification of instructors and/or therapists in the meditative movement and control groups, co-intervention, and/or quantitative data for calculating the pooled effect size of the effect of meditative movement versus control group on depression severity. In addition, given that some researchers may not have clearly described the methodology in their published papers that had been actually used in their studies, we also emailed the author to confirm the information, such as randomization procedure(s) and allocation concealment, blinding of assessor(s), and/or use of intent-to-treat analysis. Luckily, 90.9% (*n* = 10) of the authors emailed us back in response to our questions accordingly.

### 2.4. Assessment of Methodological Quality for Selected Trials

Methodological quality of the selected trials was assessed using the Physiotherapy Evidence Database (PEDro) scale [30]. The original PEDro scale consists of 11 items, including eligibility criteria, random allocation, concealment of allocation, baseline equivalence, blinding of stakeholders (participants, instructors, and assessors), retention rate of more than 85%, intention-to-treat analysis, between-group statistical comparisons, and point measures, and measures of variability. Given the fact that blinding of participants and instructors are impractical during a meditative movement intervention, these items were removed from the original scale, leading to a total of nine items. Points were only awarded if a criterion was clearly satisfied, with high scores indicating better methodological quality.

### 2.5. Data Synthesis and Analysis

Based on the random-effects model, effect sizes (standardized mean difference, SMD) across individual studies were pooled in Comprehensive Meta-Analysis Software. The forest plots were automatically generated for the severity of depression by entering the quantitative data (mean, standard deviation, and the number of participants in each group). The magnitude of SMD was classified according to the following cut-off values: (i) 0–0.19 = negligible effect, (ii) 0.2–0.49 = small effect, (iii) 0.5–0.79 = moderate effect, and (iv) 0.8 or above = large effect [31]. I-squared was also computed to determine the degree of homogeneity of effect sizes across the selected individual trials: (i) 25% = small, (ii) 50% = medium, and (iii) 75% = large. The funnel plot was used to visually assess publication bias, along with the Egger’s regression intercept test. We also calculated the odd ratio (OR) for two dichotomous outcomes (response rate and remission rate).

To decrease the unit-analysis error, if there were trials with more than one control groups, the sample size of the meditative movement group was equally divided for two comparisons, with means and standard deviations of the meditative movement group remaining unchanged [32]. For those studies with two measuring scales on the depression severity, we selected the clinician-administered scale [33]. For moderator analysis, subgroup meta-analysis and meta-regression were individually performed for categorical variables and continuous variables, based on mixed-effects models. The categorical moderators included attrition rate ≥ 15% (Yes vs. No), 100% of MDD (Yes vs. No), predominant ethnicity (Chinese/Indians vs. Caucasian/Hispanic), control type (Yes vs. No), type of meditative movement (Tai Chi/Qigong vs. Yoga), intervention duration (<12 weeks vs. ≥12 weeks), training mode (group vs. mixed), and concomitant drugs/psychotherapy (Yes vs. No). Continuous moderators included the mean age of participants and total minutes of meditative movement intervention. In our meta-analysis, subgroup analysis based on study quality was not performed because none of the selected trials scored 5 (low quality) or below.

## 3. Results

### 3.1. Trial Selection

**Figure 1:**
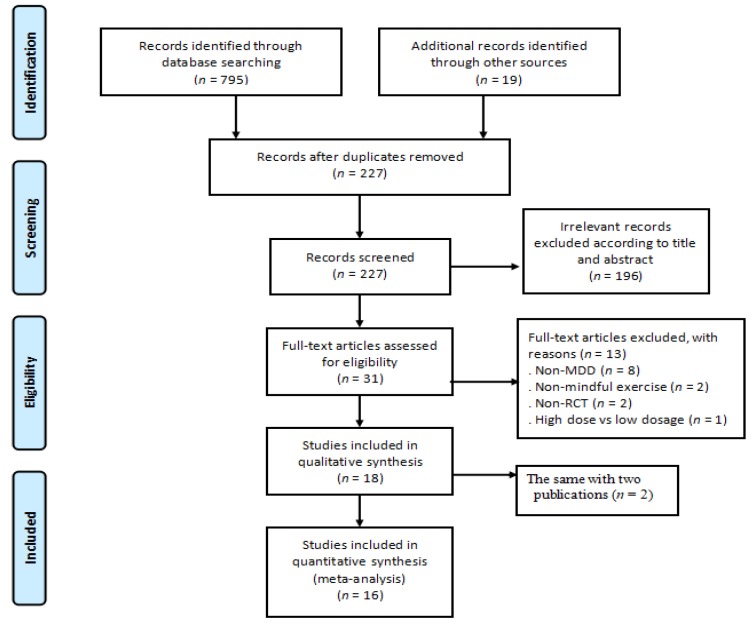
The detailed process of trial selection (MDD = major depressive disorder; RCT = randomized controlled).

Both electronic and manual searches resulted in 814 records in total. Thirty-one full-text articles were assessed according to the pre-determined inclusion criteria, leading to a final number of 16 eligible RCTs. The detailed process of trails selection is showed in Figure 1.

### 3.2. Study Characteristics 

The study characteristics of selected trials are summarized in Table 1. Eleven trials were conducted in the US [24,26,27,34,35,36,37,38,39,40,41], two in India [42,43] and China (Hong Kong), [25,28] and one in Germany [44]. All trials were published in English-language journals. Standard diagnostic criteria (DSM-IV, Structured Clinical Interview for Depression, or the Mini-International Neuropsychiatric Interview) were used to determine the eligibility of participants. Diagnostic procedures were administered by qualified assessors (psychiatrist, clinician, or trained research associate supervised by a psychiatrist). The sample size ranged from 14 to 122 (attrition rate from 5.1% to 34.2%), with the mean age ranging from 26.6 to 72.6. Only four trials did not include 100% of patients with MDD, and they were 90% [25], 81.5% [38], 75% [39], and 64.7% [40]. Female percentages in the selected trials ranged from 36.7% to 100%. Predominant ethnicities were Chinese (including Chinese Americans) [25,26,27,28], Caucasians [24,36,37,38,39,40,41,44], Indians [42,43], and Hispanic/and or black [34,35].

The duration of meditative movement intervention varied greatly, ranging from four to 12 weeks. Of the trials, only three used follow-up assessment with four weeks [36], 52 weeks [38], and 34 weeks [40]. Each training session varied greatly (20 to 210 minutes), and weekly training frequency ranged from one to six times. While Yoga was the most frequently used meditative movement intervention [35,36,37,38,39,40,41,42,43,44], Tai Chi and Qigong were only used in four trials [24,25,26,27] and one trial [28], respectively. One trial reported a combined training of Tai Chi and Yoga [34]. When meditative movement as the primary intervention, co-intervention (drug therapy and/or psychotherapy) was reported in nine trials [24,25,26,28,36,37,40,43]. Instructor-led group training was the most frequently used training mode [24,25,26,27,28,34,35,40,41,42,43,44] followed by mixed method (instructor-led group plus individual practice) in three trials [37,38,39]. Only one trial reported individual practice [36]. There were trials investigating the effects of meditative movements on depression severity (*n* = 16) [24,25,26,27,28,34,35,36,37,38,39,40,41,42,43,44], remission rate (*n* = 6) [26,27,37,40,42,43], response rate (*n* = 5) [26,27,37,40,44], and anxiety (*n* = 5) [34,35,37,38,43]. No exercise-related adverse events were reported.

### 3.3. Study Quality Assessment 

Study quality of all the selected trials is summarized in Table 2. The selected trials demonstrated fair-to-high (sum scores ranged from 6 to 9) study quality, with a mean value of 7.6 and a median of 8. It was noted that allocation concealment was not used in half of the selected trials [25,27,36,37,38,42,43,44]. Three trials did not use intention-to-treat analysis for missing data [28,36,39].

### 3.4. Effects of Meditative Movements on Dichotomous and Continuous Outcomes

Based on control type (active and passive conditions), two sub-analyses were performed to determine the effects of meditative movements on remission rate (8 arms) [26,27,37,40,42,43] and response rate (six arms) [26,27,37,40,44], respectively. Meditative movement interventions showed a significantly improved treatment remission rate (HAM-D17 = 17-item Hamilton Depression Rating Scale (HAM-D_17_ score) ≤ 7 or Quick Inventory of Depression Symptomatology-Clinician Rating (QIDS scores) ≤ 5) (OR = 6.7, 95% CI 2.38 to 18.86, *p* < 0.001) over passive control, but not active control (OR = 1.06, 95% CI 0.38 to 2.92, *p* = 0.91). Similarly, meditative movement interventions showed a significantly improved treatment response rates (over 50% improvement on the QIDS or HAM-D_17_ scores) (OR = 5.2, 95% CI 1.73 to 15.59, *p* < 0.001) over passive control, but not active control (OR = 1.58, 95% CI 0.8 to 3.13, *p* = 0.19).

**Figure 2:**
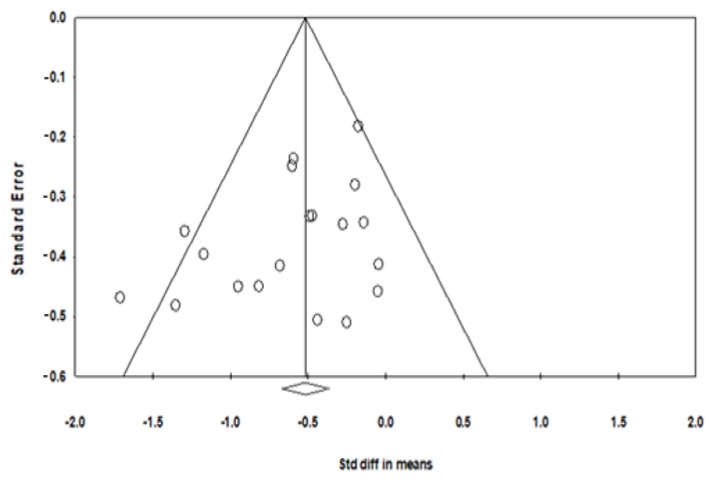
Funnel plot of publication bias for depression.

**Figure 3:**
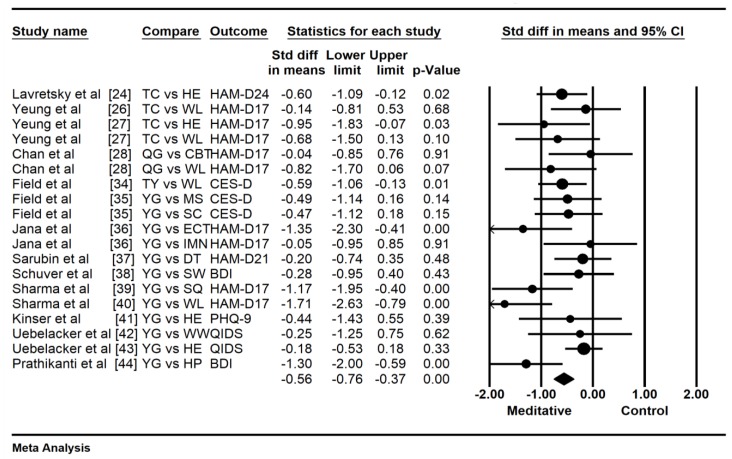
Effects of meditative movements on depression.

**Figure 4:**
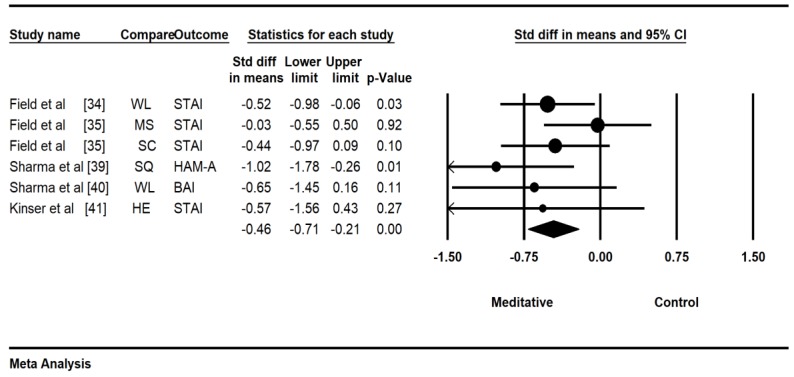
Effects of meditative movements on anxiety.

To detect the consistency of the effects of meditative movements on depression severity, a sensitivity analysis was performed by removing a trial with an outlying effect size (*SMD* = −2.37) [25], based on the visually asymmetrical Funnel plot (Figure 2) and the Egger’s Regression Test (Egger’s regression intercept = −2.172, *p* = 0.01). While this outlier was removed for further analysis, the funnel plot of remaining trials showed a symmetrical Funnel plot (Egger’s regression intercept = −1.71, *p* = 0.07). For the meta-analysis in 15 trials with 19 treatment arms, compared with the control group, the aggregated result showed a significant benefit in favor of meditative movements on depression severity (*SMD* = −0.56, 95% CI −0.76 to −0.37, *p* < 0.001, *I*^2^ = 35. 76%; Figure 3). Because the anxiety severity was not considered as the primary outcome in a small number of trials, a pooled effect size was calculated to determine the magnitude of treatment effects on the anxiety severity with no subgroup analysis of control type and other potential moderators. The results of the meta-analysis indicated a significant benefit in favor of meditative movements on anxiety severity (*SMD* = −0.46, 95% CI −0.71 to -0.21, *p* < 0.001, *I*^2^ = 1.17%; Figure 4).

### 3.5. Moderator Analysis 

There were no significant moderator effects on both categorical and continuous variables using mixed-effects model analyses (Table 3). When moderator analysis was performed using the fixed-effects model, a significant difference between two control conditions existed (Q = 4.2, df = 1, *p* = 0.04): (1) meditative movements vs. active control (*SMD* = −0.42); (2) meditative movements vs. passive control (*SMD* = −0.76). For training mode, although no significant difference was observed, there was an increasing trend in terms of the magnitude of effects of meditative movement on the depression severity: Individual (*SMD* = −0.28) < group (*SMD* = −0.55) < mixed mode (*SMD* = −0.84).

## 4. Discussion

As the first systematic review and meta-analysis synthesize the evidence of the effects of meditative movements (specifically Tai Chi, Qigong, and Yoga) on MDD, we found that meditative movements may have positive effects on the treatment of MDD, and importantly, with no occurrence of significant adverse events. This evidence suggests that there is a possibility for using these exercises as an alternative and/or augmentation approach to conventional treatments for MDD. In addition, our findings also suggests that mixed training modes may be an optimal method for treating MDD, as compared to instructor-led group practices or self-practice alone.

The potential mechanism for how meditative movements work to treat MDD symptoms remains elusive. According to the traditional Chinese medicine theories, Qi or life energy circulates via 12 main meridians (pathways) within the human body [45]. Traditional Chinese physicians believe that somatopsychic disorders occur when the flow of Qi becomes stagnant or blocked, whereas a free flowing and balanced Qi is a sign of good health and spirit [45]. Meditative movements as a mind-body healing art, are believed to cultivate the life energy and enhance its flow [45], which may potentially alleviate the progression of depressive symptoms. Qi is usually carried out by regulating breathing. Qigong, Yoga, and Tai Chi practitioners use typical breathing techniques (in a form of abdominal breathing) during the whole practice. Some studies indicated that abdominal breathing could significantly decrease negative affect and reduce cortisol levels [46]. Abdominal breathing might serve as a bridge linking the autonomic nervous system and the central nervous system to mobilize vagal activation of GABA (gamma-aminobutyric acid) pathways from the prefrontal cortex and insula, and to inhibit amygdala overactivity [47]. This might partly explain why meditative movements have a positive influence on depression.

Recently, advances in brain imaging techniques have provided another option for researchers to investigate the possible neurophsyiological mechanisms underlying the beneficial effects of meditative movements for treating MDD [48]. A neurophysiological study by Chan et al. [49] indicates that a holistic intervention (i.e., Chinese Chan-based mind-body movements, plus health education and diet modification for 10 weeks) significantly elevated left-side anterior activation (an index of positive mood) in individuals with MDD, whereas such encouraging findings were not found in either cognitive behavioral therapy nor waitlist groups. The author further explained that the significant elevation in the brain region in the holistic intervention group of MDD was positively associated with decreased depression severity, as measured by the BDI [49]. Thus, the depression-alleviated effect of meditative movements for patients with MDD may be meditated by elevating the left-side anterior activation.

In this study, we searched empirical studies published in both English and Chinese. We feel that it is important to do so because traditionally, research on Tai Chi and Qigong were predominantly performed in China and Asian countries. Surprisingly, no Chinese-language studies were found to investigate the therapeutic effects of meditative movements for MDD. The medium-to-high quality RCTs in this systematic review showed that meditative movements are a safe and effective treatment for patients with MDD. Such result is attributed to data request by contacting the original of all selected trials and to confirm the unclear report of methodological procedures in some published papers. As suggested by Chen et al. the quality of RCTs may directly affect the magnitude of the pooled effect size [50], but the procedure of data request was not employed in the recently published review that evaluates the effects of Yoga on MDD [23]. To accurately address the strengths of selected RCTs and to objectively interpret the study findings, data requests are necessary in future review studies.

Several study limitations of this systematic review should be acknowledged while interpreting our research findings. One of the most important drawbacks is that the concealment of the intervention was difficult as subjects knew whether they received meditative movement intervention, even though a centralized randomization procedure was used. This might lead to subjectivity and expectation bias by the participants. Second, allocation concealment in half of the selected trials was absent in this systematic review. Such inadequate concealment of allocation was associated with exaggerated estimates of meditative movement treatment benefits for MDD [51]. Third, meditative movements were not offered as mono-therapy in half number of studies, but as adjunctive treatments to existing interventions (drug or psychological therapy). It may be difficult to conclude whether the positive outcomes were attributed to the meditative movement alone, a synergetic intervention effect, or to the conventional treatment received by the patients. This was also confounded by the fact that a variety of interventions were received by control groups. When sub-analysis was performed on two dichotomous outcomes using six trials, meditative movements did not have significant effects on remission rate and response rate when more stringent (active) controls were used. Nevertheless, results from our overall analysis provide support for meditative movements as an adjunctive treatment or as a mono-therapy for reducing depressive symptoms. Fourth, the duration of the meditative movement interventions varied a greatly across included studies, leading to difficulties in recommending an optimal dose of intervention. It is possible that in most studies, participants had attained the minimal duration needed to obtain the psychological benefits. Fifth, because the follow-up period varies greatly in a small number of trials on the outcomes, meta-analysis was not performed to investigate the long-term effects of meditative movements for MDD in this present review. Sixth, the findings of this study may not apply to patients with very severe depression (HAMD score ≥23). These patients are usually excluded in the selected studies since many ethics committees require conventional treatment, rather than meditative movements as interventions for this group, considering the severity of their illnesses. Thus, interpretation of this systematic review is limited to the immediate effects of meditative movements on alleviating MDD symptoms.

## 5. Conclusions

This systematic review, based on the existing literature, suggests that meditative movements may be effective interventions to alleviate MDD symptoms. Emphasizing the therapeutic effects of meditative movements for treating MDD symptoms is critical because it may provide a useful option to existing mainstream treatments (drug therapy and psychotherapy) for patients with MDD. Given the fact that meditative movements are safe and easily accessible, clinicians may consider recommending meditative movements for patients of MDD. It must be addressed that significant methodological limitations were found in most of the empirical studies to date, which may have impacted the interpretation of these findings. More randomized controlled trials with rigorous research design are warranted to establish the therapeutic effects of meditative movements for MDD, and its potential use for prevention and as an adjunctive treatment for MDD.

## Figures and Tables

**Table 1:** Characteristics of all randomized controlled trials.

Author, Country	Participant Characteristics						Intervention Protocol					Outcome Measured and Safety
	Diagnostic Criteria, Assessor	N (AT)	MDD	Female	Predominant Ethnicity	Age (Year)	Weekly Dosage (Type of ☯ and ○, Qualified Instructor [Yes/No])	TTT (Min)	Ind or Grp	Duration (Weeks),FU	Drug and/or PSY	1 = Primary and 2 = Secondary Outcome (Measuring Instrument); Adverse Event
Lavretsky et al. (2011) [24] USA	DSM-IV, a psychiatrist	73 (6.8%)	100%	61.6%	74% Caucasian	70.57	☯ (36): 1 × 120 min/ week (Tai Chi, yes) ○ (37): 1 × 120 min/ week (HE, yes)	1200	Grp	10, No	Yes	1. Depression severity (HAM-D_24_); No
Chou et al. (2004) [25] China	DSM-IV, a psychiatrist	14 (0%)	90%	50%	100% Chinese	72.6	☯ (7): 3 × 45 min/week (Tai Chi, yes) ○ (7): waitlist	1620	Grp	12, No	Yes	1. Depression severity (CES-D); No
Yeung et al. (2012) [26] USA	DSM-IV, a psychiatrist	39 (5.1%)	100%	77%	100% Chinese American	55	☯ (26):2 × 60 min/week (Tai Chi, yes); ○ (13): waitlist	1440	Grp	12, No	Yes	1. Remission rate (HAM-D_17_ scores ≤ 7), Response rate (≥ 50% improvement on the HAM-D_17_ score) and depression severity (HAM-D_17_); No
Yeung et al. (2017) [27] USA	DSM-IV, a psychiatrist	67 (25.3%)	100%	72%	100% Chinese American	54	☯ (23):2 × 60 min/week (Tai Chi, yes); ○ (22):2 × 60 min × 2/week (HE, yes); ◎ (22): waitlist	1440	Grp	12, 12	None	1. Remission rate (HAM-D_17_ ≤ 7), Response rate (≥ 50% improvement on the HAMA_17_ score), and depression severity (HAM-D_17_ and BDI); No
Chan et al. (2012) [28] China	DSM-IV, a psychiatrist	75 (33.3%)	100%	80%	100% Chinese	46.48	☯ (25):1 × 90 min/week (Qigong, yes); ○ (25):1 × 90 min/week (CBT, yes); ◎ (25): waitlist	900	Grp	10, No	Yes	1. Depression severity (HAM-D_17_ and BDI); No
Field et al. (2013) [34] USA	DSM-IV, a RA supervised by a psychiatrist	92 (18.5%)	100%	100%	57% Hispanic, 40% Black	26.6	☯ (46):1 × 20 min/ week (Tai Chi +Yoga, yes); ○ (46): waitlist	240	Grp	12, No	None	1. Depression severity (CES-D), 2. Anxiety (State-Trait Anxiety Inventory); No
Field et al. (2012) [35] USA	SCID, a RA supervised by a psychiatrist	84 (11%)	100%	100%	38% Hispanic, 40% Black, 12% Caucasian	28.57	☯ (28):1 × 20 min/week (Yoga, yes); ○ (28):1 × 20 min/week (massage, yes); ◎ (28): standard care	240	Grp	12, No	None	1. Depression severity (CES-D), 2. Anxiety (State-Trait Anxiety Inventory); No
Janakiramaiah et al. (2000) [42] India	DSM-IV, a psychiatrist	45 (0%)	100%	44.4%	100% Indian	38.7	☯ (15) :4–6 × 45 min/week (Yoga, yes); ○ (15): 3 times/week Electroconvulsive therapy; ◎ (15):150 mg/day (imipramine)	900	Grp	4, No	No in ☯	1. Remission rate (HAM-D_17_ score ≤ 7), Depression severity (HAM-D_17_ and BDI); No
Sarubin et al. (2014) [44] Germany	DSM-IV, a psychiatrist	53 (0%)	100%	39.5%	100% Caucasian	40.25	☯ (22):1 × 60 min/week (Yoga, yes); ○ (31):300 mg/day (QXR) or 10 mg/day (ESC)	300	Grp	5, No	No in ☯	1. Response rate (≥ 50% improvement on the HAMA_21_ score) and depression severity (HAM-D_21_); No
Schuver et al. (2016) [36] USA	SCID, RAs supervised by a psychiatrist	40 (15%)	100%	100%	80% Caucasian	42.68	☯ (20): 2 × 60–75 min/week (Yoga, no) + 15 min (weekly telephone counselor); ○ (20): 2 × 65 min/week (self-walking) + 15 min (weekly telephone counselor)	1740	Ind	12, 4	Yes	1. Depression severity (BDI); No
Sharma et al. (2005) [43] India	DSM-IV, two psychiatrists	30 (0%)	100%	36.7%	100% Indian	31.77	☯ (15):3 × 30 min/week (Yoga, yes); ○ (15): sitting quietly	720	Grp	8, No	Yes	1. Remission rates (HAM-D_17_ score ≤ 7). depression severity (HAM-D_17_), 2. Anxiety (HAM-A_17_); No
Sharma et al. (2017) [37] USA	DSM-IV, a psychiatrist	25 (12%)	100%	72%	92% Caucasian	37.19	☯ (13): 6 × 210 min for week 1 + 1 × 90 min for week 2–8 (Yoga, yes) + 20–25 min daily home practice; ○ (12): waitlist	1890	Mixed	8, No	Yes	1. Remission rate (HAM-D_17_ score ≤ 7), response rate (≥ 50% improvement on the HAMA_17_ score and Depression severity (HAM-D_17_ and BDI), 2. Anxiety (Beck Anxiety Inventory); No
Kinser et al. (2013) [38] USA	MINI, a board-certified clinician	27 (33%)	81.5%	100%	63% Caucasian	43.26	☯ (15): 1 × 75 min/week + home practice (Yoga, yes); ○ (12): 1 × 75 min/week (HE, yes)	600	Mixed	8, 52	Yes	1. Depression severity (Patient Health Questionnaire-9), 2. Anxiety (State Trait Anxiety Inventory); No
Uebelacker et al. (2016) [39] USA	DSM-IV, psychiatrists	20 (10%)	75%	100%	75% Caucasian	28.4	☯ (12):1 × 75min × 1/week + home practice (Yoga, yes); ○ (8): 1 × 75 min/week (WW, yes)	675	Mixed	9, No	None	1. Depression severity (QIDS); No
Ubelacker et al. (2017) [40] USA	SDM-IV, two psychologists	122 (14.8%)	64.7%	84.4%	84% Caucasian	46.5	☯ (63): 2 × 80 min/week (Yoga, yes); ○ (59): 1–2 × 60min/week (HE, yes)	1600	Grp	10, 34	Yes	1. Remission rate (QIDS scores ≤ 5), response rate ≥ 50% improvement on the QIDS), and depression severity (the QIDS); No
Prathikanti et al. (2017) [41] USA	MINI, a psychiatrist	38 (34.2%)	100%	68%	58% Caucasian	43.4	☯ (20):2 × 90min/week (Yoga, yes); ○ (18):2 × 90 min/week (Education on Yoga history& philosophy, yes)	1440	Grp	8, No	None	1. Depression severity (BDI); No

Note: N = sample size; AT = attrition rate; MDD = major depressive disorder; RA = research associate; y = year; Ind = individual training; Grp = group training; FU = follow-up; PSY = psychotherapy; DSM-IV = Diagnostic Statistical Manual of Mental Disorders, Fourth Edition; SCID = Structured Clinical Interview for Depression; MINI = Mini-International Neuropsychiatric Interview; HAM-D_24_ = 24-Item Hamilton Depression Rating Scale; CES-D = Epidemiological Studies Depression Scale; HAM-D_17_ = 17-item Hamilton Depression Rating Scale; BDI = Beck Depression Inventory; HAM-D_21_ = 21-item Hamilton Depression Rating Scale; BAI = Beck Anxiety Inventory; QIDS = Quick Inventory of Depression Symptomatology-Clinician Rating; HAM-A_17_ = 17-item Hamilton Anxiety Rating Scale; TTT = Total training time; QXR = Quetiapine fumarate extended release; ESC = escitalopram; BAI = Beck Anxiety Inventory; HE = health education; CBT = cognitive behavioral therapy; WW = wellness workshop; AE = adverse event; ☯: Meditative movement intervention; ○: Control group 1; ◎: Control group.

**Table 2:** Study quality assessment of all selected trials.

Reference	Item 1	Item 2	Item 3	Item 4	Item 5	Item 6	Item 7	Item 8	Item 9	Sum Score
Lavretsky et al. (2011) [24]	1	1	1	1	1	1	1	1	1	9/9
Chou et al. (2004) [25]	1	1	0	1	1	1	1	1	1	8/9
Yeung et al. (2012) [26]	1	1	1	1	1	1	1	1	1	9/9
Yeung et al. (2017) [27]	1	1	0	1	0	0	1	1	1	6/9
Chan et al. (2012) [28]	1	1	1	1	1	0	0	1	1	7/9
Field et al. (2013) [34]	1	1	1	1	1	0	1	1	1	8/9
Field et al. (2012) [35]	1	1	1	1	1	1	1	1	1	9/9
Janakiramaiah et al. (2000) [42]	1	1	0	1	1	1	1	1	1	8/9
Sarubin et al. (2014) [44]	1	1	0	1	0	1	1	1	1	7/9
Schuver et al. (2016) [36]	1	1	0	1	1	0	0	1	1	6/9
Sharma et al. (2005) [43]	1	1	0	1	0	1	1	1	1	7/9
Sharma et al. (2017) [37]	1	1	0	1	1	1	1	1	1	8/9
Kinser et al. (2013) [38]	1	1	0	1	1	0	1	1	1	6/9
Uebelacker et al. (2016) [39]	1	1	1	1	1	1	0	1	1	8/9
Ubelacker et al. (2017) [40]	1	1	1	1	1	1	1	1	1	8/9
Prathikanti et al. (2017) [41]	1	1	1	1	1	0	1	1	1	8/9

Note: Item 1 = eligibility criteria; Item 2 = randomization; Item 3 = concealed allocation; Item 4 = similar baseline; Item 5 = blinding of assessors; Item 6 = more than 85% retention; Item 7 = missing data management (intent-to-treat analysis); Item 8 = between-group comparison; Item 9 = point measure and measures of variability; 1 = explicitly described and present in details; 0 = absent, inadequately described, or unclear.

**Table 3:** Moderator analysis for depression severity.

Categorical Moderator	Outcome	Level	No. of Studies/Comparisons	Hedges’ g	95% Confidence Interval	*I*^2^, %	Test for between-Group Homogeneity		
							*Q*-Value	df(Q)	*p*-Value
Attrition Rate ≥15%	Depression	Yes	8	−0.59	−0.96 to −0.32	5.42%	0.33	1	0.57
		No	11	−0.52	−0.77 to −0.26	48.39%			
100% of MDD	Depression	Yes	16	−0.62	−0.83 to −0.42	35.21%	2.37	1	0.12
		No	3	−0.23	−0.68 to 0.22	0%			
Predominant Ethnicity	Depression	Caucasian/Hispanic	11	−0.54	−0.78 to −0.29	41.18%	0.00	1	0.97
		Chinese/Indians	8	−0.62	−0.97 to −0.28	33.3%			
Control Type	Depression	Active	13	−0.45 ^☯^	−0.67 to −0.23	11.8%	2.76	1	0.15 ^☯^
		Passive	6	−0.79 ^☯^	−1.13 to −0.46	51.05%			
Mindfulness Type	Depression	Taichi/Qigong	6 ^※^	−0.64	−1.04 to −0.23	44.07%	0.02	1	0.87
		Yoga	12 ^※^	−0.7	−1.35 to −0.04	72.83%			
Intervention Duration	Depression	<12 weeks	12	−0.61	−0.87 to −0.35	56.14%	0.3	1	0.59
		≥12 weeks	7	−0.5	−0.82 to −0.17	0%			
Training mode	Depression	Group	15	−0.55 ^®^	−0.77 to −0.33	32.3%	0.71	1	0.4
		Mixed	3	−0.84 ^®^	−1.47 to −0.21	63.21%			
Drug and/or psychotherapy	Depression	Yes	9	−0.52	−0.80 to −0.23	49.94%	0.21	1	0.65
		No	10	−0.61	−0.89 to −0.33	19.19%			
**Continuous Moderator**	**Level**	**No. of Studies/Comparisons**	**β**	**95% Confidence Interval**		***Q*-Value**		***df***	***p***
Mean age	Depression	19	0.00292	−0.00923 to 0.01506		0.22		1	0.63
Total minutes in practice	Depression	19	−0.00003	−0.0003 to −0.22368		0.05		1	0.82

☯: When the fixed-effects model was set, a significant difference between two different control types existed (*Q* = 4.2, *df* = 1, *p* = 0.04), meditative movement vs. passive control (*SMD* = −0.76) and meditative movement vs. active control (*SMD* = −0.42); ※: A trial by Field [34] used a mixed meditative movement of Tai Chi and Yoga, so it was not included for data synthesis and the total treatment arms was 18; ®: A trial by Sshuver et al. [36] used the individual training mode (DVD-guided, self-practice) found a treatment effect (*SMD* = 0.28). Thus, although no significant differences among the three training modes, the magnitude of the treatment effects showed an increased trend: individual (*SMD* = 0.28) < group (*SMD* = 0.55) < Mixed (*SMD* = 0.84).
